# Highly diverse root endophyte bacterial community is driven by growth substrate and is plant genotype-independent in common bean (*Phaseolus vulgaris* L.)

**DOI:** 10.7717/peerj.9423

**Published:** 2020-06-26

**Authors:** Aarón Barraza, Juan Carlos Vizuet-de-Rueda, Raúl Alvarez-Venegas

**Affiliations:** 1 Programa de Agricultura en Zonas Áridas, CONACYT-CIBNOR, Centro de Investigaciones Biológicas del Noroeste, La Paz, Baja California Sur, México; 2 Unidad Irapuato, Centro de Investigación y de Estudios Avanzados del Instituto Politécnico Nacional, Irapuato, Guanajuato, Mexico

**Keywords:** Bacterial community structure, Growth substrates, Next-generation sequencing, Root inner bacteria, Root genotype

## Abstract

The common bean (*Phaseolus vulgaris* L.) is the most important grain legume in the human diet with an essential role in sustainable agriculture mostly based on the symbiotic relationship established between this legume and rhizobia, a group of bacteria capable of fixing atmospheric nitrogen in the roots nodules. Moreover, root-associated bacteria play an important role in crop growth, yield, and quality of crop products. This is particularly true for legume crops forming symbiotic relationships with rhizobia, for fixation of atmospheric N_2_. The main objective of this work is to assess the substrate and genotype effect in the common bean (*Phaseolus vulgaris* L.) root bacterial community structure. To achieve this goal, we applied next-generation sequencing coupled with bacterial diversity analysis. The analysis of the bacterial community structures between common bean roots showed marked differences between substrate types regardless of the genotype. Also, we were able to find several phyla conforming to the bacterial community structure of the common bean roots, mainly composed by *Proteobacteria*, *Actinobacteria*, *Bacteroidetes*, *Acidobacteria*, and *Firmicutes*. Therefore, we determined that the substrate type was the main factor that influenced the bacterial community structure of the common bean roots, regardless of the genotype, following a substrate-dependent pattern. These guide us to develop efficient and sustainable strategies for crop field management based on the soil characteristics and the bacterial community that it harbors.

## Introduction

Plants have evolved with an overabundance of microorganisms having important functions supporting plant growth and health. Since the first agricultural revolution, humans have domesticated a considerable number of plant species with desirable traits, and the careful breeding of high yielding genotypes. Furthermore, the transition from food gathering to farming has hindered beneficial interactions between plants and microbes. For example, constant nitrogen fertilization has resulted in the loss of soil microbial diversity due to the evolution of less-mutualistic rhizobia (Pérez-Jaramillo, Mendes & Raaijmakers, 2016). Nonetheless, plants host a high diversity of microorganisms known as the plant microbiota (which encompass all microorganisms) (Bulgarelli et al., 2013). Particularly, land plants host abundant and diverse microbial communities in the rhizosphere, the area surrounding the plant roots (McNear, 2013). The plants and microbes may have commensalistic, mutualistic, or even pathogenic relationships, and some rhizobacteria enter the root and live as endophytes (Buée et al., 2009). Consequently, rhizobacteria have been linked to soil-borne diseases, resistance to abiotic stresses, the facility of nutrient acquisition, and they are considered a crucial aspect of the plant’s performance, growth, survival, and also, are directly influenced by plant root exudates (rhizodeposition), mucilage, and cellular debris that influences the chemical and physical composition of the rhizosphere and provides signaling molecules and organic substrates for microbial growth (Wu et al., 2015; Bulgarelli, 2018).

Also, the genotype of the plant and the type of soil (its edaphic characteristics), influence the function and metabolic activities of rhizobacteria, as well as the structure of their communities (Shang et al., 2016). Indeed, the particular bacterial communities present in the rhizosphere are influenced by the plant species, in a host-dependent way, and even by the type of cultivars or inbred lines within a single species (Peiffer et al., 2013; Pérez-Jaramillo, Mendes & Raaijmakers, 2016; Pérez-Jaramillo et al., 2019). Moreover, several studies have shown that breeding and domestication have altered the interactions between crops and beneficial microorganisms, such as symbiotic rhizobia associations of domesticated legumes present a reduced diversity when they are compared to wild legumes (Mutch & Young, 2004; Lynch et al., 2004). Also, Brisson et al. (2019) have shown that advances in hybrid development have had a reduction of rhizosphere microbial communities diversity and also a decrease of network assembly and that the changes in microbial community recruitment associated with modern breeding may have been higher than those associated with domestication. Root microbiome composition is also affected by soil type, soil moisture, soil structure, pH, salinity, and soil organic matter and exudates. It is essential to bring microbial innovations into practice owing to the exciting functional potential of the plant microbiome, as well as to the new challenges in crop production (Compant et al., 2019).

Crop diversification, intercropping, and other cultural practices have been used for sustainable agricultural production. In recent years, plants have been engineered to, for example, create enhanced crops. Genetically modified plants can have many valuable attributes for crop improvement, such as disease or pest resistance, herbicide tolerance, improved nutritional value, or more significant biofuel potential (Stuart et al., 2010; Robertson-Albertyn et al., 2017; Garcia Ruiz, Knapp & Garcia-Ruiz, 2018; Wang et al., 2019a). Several studies indicate that while transgenic plants may affect the rhizosphere and endophytic microbiome, it is not always the expected result, such as in transgenic rice and transgenic poplars (Wang et al., 2019a, 2019b). Understanding the interactions between bacterial communities, their host plants, and soil type will be of great importance in future plant breeding programs and plant biotechnology. This knowledge should facilitate the advancement of novel crop and plant production systems to successfully deal with harsh environmental conditions, the stress imposed by pests and pathogens, and limited resource availability. Given the ever-increasing world population, this knowledge should be of value in our ability to sustain food production (Martin, Uroz & Barker, 2017).

The common bean (*Phaseolus vulgaris* L.) is the most important grain legume in the human diet (http://www.fao.org/3/a-av015e.pdf) and has a critical role in sustainable agriculture. This role is based on the symbiotic relationship between legumes and rhizobia, a group of bacteria capable of fixing atmospheric nitrogen in the roots nodules (Crespi & Frugier, 2008). Also, *P. vulgaris* is recalcitrant to in vitro induction of somatic embryogenesis and regeneration. However, composite common bean plants, with wild-type shoots and transgenic hairy roots (composite plants), have been successfully developed (Estrada-Navarrete et al., 2006, 2007). We used this approach to compare the effect of common bean (*P. vulgaris* L.) root genotypes (wild-type and transgenic) and substrate type (soil and vermiculite) on the assembly of their inner bacterial communities through 16S rDNA (V3 region) for bacterial diversity analysis. Our main goal was to establish whether different common bean root genotypes associate, or not, with different bacterial communities and to show the effect of transgenic hairy roots on their inner bacterial communities when compared to common bean roots wild-type grown in different substrate types.

## Materials and Methods

### Plant material and growth conditions

*Phaseolus vulgaris* L. cultivar Negro Querétaro seeds were surface sterilized with 2% (v/v) sodium hypochlorite for fifteen minutes. Then, seeds were rinsed five times with sterile water, placed in sterile plates containing wet paper towels, covered with aluminum foil, and placed for 3 days at 28 °C in a Percival growth chamber (16 h light; 8 h dark cycle; Percival Scientific, Perry, IA, USA; Barraza et al., 2018). At the end of this time, the seedlings were planted in pots containing vermiculite (is an inert and nutrient lack substrate) or potting soil (is an acid substrate with a minimal nitrogen content) (brand “Vigoro”; Sulfatos y Derivados S.A., México) and returned to the growth chamber.

### Bacterial strains

Competent *Agrobacterium rhizogenes* strains K599: NCPPB 2659 containing either the PvTRX1h-asRNA silencing vector (also known as PvTRX1h-RNAi), or the pK7Neg control expression vector were used to transform the roots of common bean (*Phaseolus vulgaris* L.). Control transformations were performed with *A. rhizogenes* K599 (with no vector). These bacterial strains have been previously described by Barraza et al. (2018). Wild-type common bean plants were not treated with *A. rhizogenes*.

### Construction of transgenic hairy root genotypes

*A. rhizogenes* K599 (without any vector), *A. rhizogenes* K599 containing the PvTRX1h-asRNA (RNAi) silencing vector, and the pK7Neg control expression vector were used to generate transgenic hairy roots as previously described (Barraza et al., 2015, 2018). In brief, an *A. rhizogenes* bacterial suspension was collected in a syringe (Estrada-Navarrete et al., 2007). Next, the cotyledonary node of 6-day-old seedlings was slightly wound with the needle tip (four times at different positions around the node) and ~5 × 10^6^ cells/μL of the inoculum were injected into the wound (Estrada-Navarrete et al., 2007; Barraza et al., 2018). Fourteen days after infection with *A. rhizogenes*, transgenic hairy roots were well developed, and untransformed non-fluorescent hairy roots were cut off (Fig. S1). The primary root was removed by cutting the stem below where the hairy roots emerged (Estrada-Navarrete et al., 2007) and the obtained composite plants were placed in 2.3-L pots containing either potting soil or vermiculite. Common bean roots wild-type (WT) genotype were not inoculated with *A. rhizogenes* K599. No bacterial inoculum was added neither the beginning nor during the study in any of the experimental conditions (WT or transgenic roots K599, pK7Neg, and RNAi) in soil or vermiculite. All plants were watered every other day with a Broughton and Dilworth (B&D) solution for 15 days (Barraza et al., 2015, 2018).

### Experimental design

**Figure 1:**
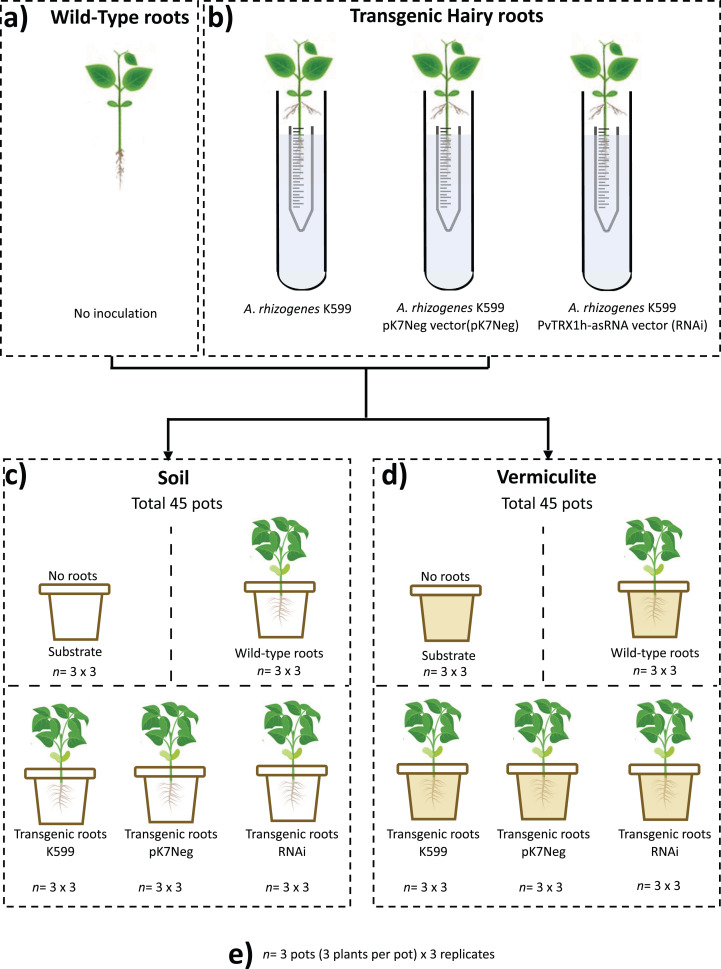
Schematic experimental representation applied in this study. For three biological replicates by three experimental replicates (*n* = 3 × 3). (A) Wild-type common bean plants; (B) composite common bean plants (untransformed shoot with transgenic hairy roots); (C) composite common bean plants, wild-type common bean plants in soil substrate, and unplanted soil substrate; (D) composite common bean plants, wild-type common bean plants in vermiculite substrate, and unplanted vermiculite substrate; (E) experimental replicates criteria.

A two-factorial nested (substrate and genotype) design (with five conditions: unplanted substrate, wild-type roots, transgenic roots K599, transgenic roots pK7Neg, and transgenic roots RNAi) was used. Three plants represented one biological replicate per experimental replicate for both common bean composite plants (with transgenic hairy roots) and for common bean wild-type plants (roots without any genetic modification). Three biological replicates were employed per experimental replicate, and three experimental (*n* = 3) replicates were conducted (Fig. 1). In total 45 pots were employed per substrate (soil and vermiculite). For both substrates, we used 90 pots, of which 30 experimental samples were processed for total DNA extraction for next-generation sequencing.

### Extraction of total DNA

At harvest after 15 days, the plants of each pot (Fig. 1) were removed, and the substrate (vermiculite or soil) shaken off from the roots. The root system was rinsed with sterile de-ionized water. The WT roots and transgenic roots were collected, pooled, frozen in liquid nitrogen, and stored at −80 °C. All experiments were performed in triplicate per treatment (each replicate containing fully grown hairy roots samples from three different pots per treatment). Samples (5 g) of the unplanted substrate (soil or vermiculite) were also collected and stored at −80 °C.

The total DNA from the roots and unplanted growth substrate was extracted (transgenic hairy roots, WT roots, and unplanted substrates) according to the procedure of Caamal-Chan et al. (2019) with some modifications. In brief, roots or substrates were ground in liquid nitrogen. Five grams of each sample (roots or unplanted growth substrates) were transferred to 15-mL tubes and suspended in 10 mL of ice-cold lysis buffer (2% CTAB (w/v), 1.4 M NaCl, 20 mM EDTA, 100 mM Tris-HCl pH 8.0, 0.2% (v/v) β-mercaptoethanol, and proteinase K) (Chen et al., 2015; Caamal-Chan et al., 2019). The samples (roots or unplanted growth substrates) were incubated at 50 °C for 30 min with occasional shaking; then, they were incubated for 72 h at room temperature and were centrifugated for 2 min at 5,000×*g*’s in a microcentrifuge; the supernatant was transferred to a new 15 mL tube. For DNA extraction, one volume of phenol:chloroform:isoamyl alcohol (25:24:1) was added, then the samples were centrifugated for 15 min at 12,000×*g*’s. After the ethanol precipitation, DNA was resuspended in 100 µL of TE buffer (pH 8.0). The total DNA was treated with RNase A (10 mg/mL¸ Promega, Madison, WI, USA) at 37 °C for 30 min. DNA integrity was analyzed by agarose gel electrophoresis and its purity (λ 260 nm/280 nm ratio) and quantity assessed with a NanoDrop 2000 spectrophotometer (Thermo Fisher Scientific, Waltham, MA, USA). All DNA samples were stored at −20 °C.

### PCR amplification, sequencing, and community analyses

The V3 region of the bacterial 16S rDNA was amplified by PCR (35 cycles) using the primers V3-338f and V3-533r with Illumina adapters and sample-specific tags; indices were also added, following the manufacturer’s recommendations (Illumina, San Diego, CA, USA). The V3 amplicons were quantified using a Qubit (Thermo Fisher Scientific, Waltham, MA, USA) for equimolar sample pooling (1.5 pM). Sequencing reads were generated using the 2 × 150 (300 cycles) for the base-read length chemistry of the Illumina MiniSeq platform. After quality filtering (*Q* ≥ 33) using the modified-Mott algorithm, the reads were pair-end assembled in BBmerge (Ewing et al., 1998; Ewing & Green, 1998; Kearse et al., 2012; Bushnell, Rood & Singer, 2017). The paired-end-merged reads were then de novo clustered with the Geneious Assembler with a minimum identity of 98%, and both contigs and unassembled reads were compared against the non-redundant nucleotide collection (“*nr*/*nt*”) GenBank database using Megablast (Randle-Boggis et al., 2016). Megablast results were used to create a curated database specific for each substrate and genotype (Soil: 1-3; Vermiculite: 1-3; WT roots in soil: WTs1-3; WT roots in vermiculite: WTv1-3; K599 roots in soil: K599s1-3; K599 roots in vermiculite: K599v1-3; pK7Neg roots in soil: pK7NegS1-3; pK7Neg roots in vermiculite: pK7NegV1-3; *PvTRX1h*-*asRNA* roots in soil: RNAiS1-3; *PvTRX1h-asRNA* roots in vermiculite: RNAiV1-3; see Fig. 1). The sequence reads of each sample were compared with the created database using Sequence Classifier, with a minimum of 99% identity for species’ taxonomic assignation, to finally obtain the table of operational taxonomic units (OTUs) in Geneious Prime v2019.2.1 (www.geneious.com). For data normalization, the frequency of best hits to each individual taxon for each metagenome was divided by the total number of hits per sample. We estimated the Chao1, Shannon, and Simpson (alpha) diversity indices with the package “vegan” and carried out the inter- and extrapolation analysis for such diversity indices with “iNEXT” function (order (*q*) set to 0, 1, and 2, respectively) of the package ‘iNEXT’. PERMANOVA statistical analysis was performed with the “adonis” function of the package “iNEXT” (Oksanen et al., 2014; Hsieh, Ma & Chao, 2016; Junqueira et al., 2017), and one-way ANOVA tests were carried out followed by Tukey post hoc test with the “TukeyHSD” function. Bray-Curtis distance was calculated using the “vegdist” function; as well as non-metric multidimensional scaling analysis, with the “metaMDS” function, coupled with a constrained correspondence analysis (CCA) and a distance-based redundancy analysis (dbRDA), with the “capscale” and “dbrda” functions, respectively, of the package “vegan” (Oksanen et al., 2014; Coleman-Derr et al., 2016; Castañeda & Barbosa, 2017; Pavloudi et al., 2017). The differentially abundant OTUs (DAOTUs; acquired with the DESeq2 package) as a function of root genotype (WT, K599, pK7Neg, and RNAi) were determined by fitting a generalized linear model (GLM) with a negative binomial distribution (Love, Huber & Anders, 2014). Likelihood ratio tests and contrast analyses were performed on the fitted GLM to identify DAOTUs. The OTUs counts from WT roots were used as the control, and compared with those of the transgenic roots (K599, pK7Neg, and RNAi) by contrast analyses. The significance levels were adjusted using the Benjamin-Hochberg false discovery rate correction (*P* < 0.05) (Poudel et al., 2019). General community profiles were constructed using OTUs labeled at the phylum level and visualized in a bar plot graph.

## Results

### Next-generation sequencing metrics and overall bacterial community structures in common bean roots

From all samples sequenced, 2,063,426 reads were generated, and after processing, we held with 795,463 high-quality pair-end-assembled reads, we analyzed only those 645,916 reads that could be assigned to bacteria, based on comparisons with the entries in the non-redundant GenBank nucleotide database. From all reads, 18.3% were excluded due to an apparent origin from eukaryotes and 0.5% because they could not be assigned to any taxon (Fig. S2), and the mean value of Good’s coverage for all sample reads was 99.3% ± 0.46% (sequencing effort). Of the bacterial reads, we obtained 13,793 OTUs by similarity clustering at 99% nucleotide identity and 9,711 OTUs after excluding singletons within each sample. The latter’s occurrence and prevalence were used after rarefaction sampling curves (all OTUs and at Genus taxonomic rank) to calculate and develop the graphical and statistical analysis the bacterial alpha- and beta-diversity (Fig. S3A).

**Figure 2:**
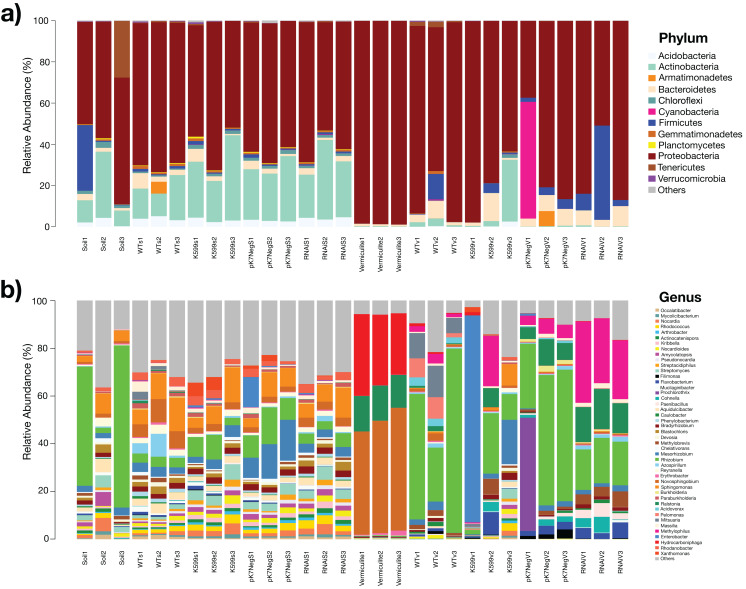
Profiles of the bacterial communities in soil and vermiculite and associated with roots of common bean (*P. vulgaris* L.) at the phylum and genus level, based on operational taxonomic units (OTUs). (A) Bacterial community profiles at the phylum level. (B) Bacterial community profiles at the genus level. OTUs with relative sequence abundances <1% are summed as “others” (Soil1-3, without plants; Vermiculite1-3, without plants; WTs1-3, wild-type roots grown in soil; WTv1-3, WT roots grown in vermiculite; K599s1-3, K599 roots grown in soil; K599v1-3, K599 roots grown in vermiculite; pK7NegS1-3, pK7Neg roots grown in soil; pK7NegV1-3, pK7Neg roots grown in vermiculite; RNAiS1-3, *PvTRX1h*-*asRNA* roots grown in soil; RNAiV1-3, *PvTRX1h-asRNA* roots grown in vermiculite).

The resulting OTUs abundance of this study revealed an unexpectedly high diversity of bacteria that assemble the bacterial communities inside the roots of the common bean for all genotypes. At the phylum level, *Proteobacteria* (55.6–98.72%) was the most abundant phylum for all samples, followed by *Actinobacteria* (0.17–27.11%), *Firmicutes* (0.02–26.8%), *Bacteroidetes* (0.95–7.39%), *Acidobacteria* (0.007–4.44%), and *Tenericutes* (0–9.44%) (Fig. 2A). The *Proteobacteria* in the soil was less abundant than in the vermiculite and there were more *Actinobacteria* in soil than vermiculite. The most abundant genera were *Rhizobium* (0.33–47.23%), *Novosphingobium* (0.30–47.15%), *Ralstonia* (0.01–13.96%), *Enterobacter* (0–29.64%), *Methylophilus* (0.05–18.21%), *Sphingomonas* (0.04–8.29%), *Hydrocarboniphaga* (0.002–27.8%), *Mesorhizobium* (0.08–10.35%), *Streptomyces* (0.0008–3.14%), *Flavobacterium* (0.01–4.18%), *Bradyrhizobium* (0.38–2.04%), and *Methylobrevis* (0.006–3.61%) (Fig. 2B). Based on the relative abundance of the bacteria from soil and vermiculite conditions, the bacterial communities present were taxonomically diverse at the genus level. Rhizobia were also more abundant when the common bean roots were raised in vermiculite than soil. A large proportion of bacteria in the substrates were of low abundance (<1%) and thus assigned as “others” (Fig. 2B).

### Estimations of bacterial diversity in common bean roots

**Figure 3:**
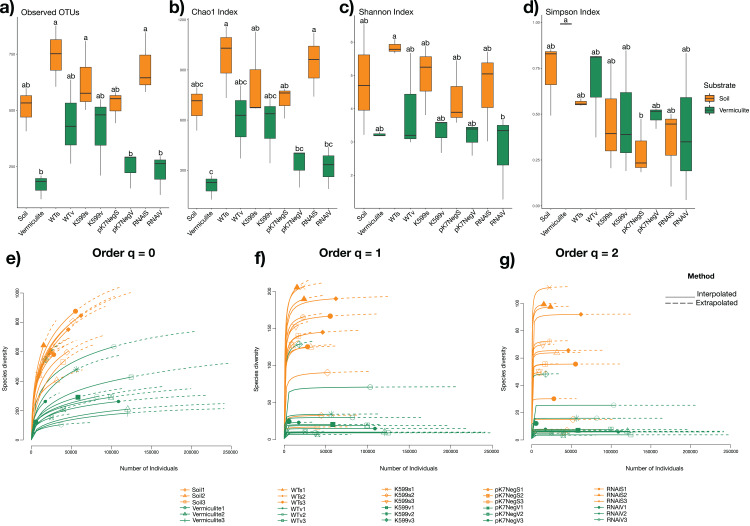
Alpha diversity of bacterial communities in soil and vermiculite and associated with roots of common bean (*P. vulgaris* L.). (A) Observed OTUs, (B) Chao1 index, (C) Shannon Index, (D) Simpson index, (E) Chao1 index estimations (*q* = 0), (F) Shannon index estimations (*q* = 1), (g) Simpson index estimations (*q* = 2) through rarefaction (interpolation) and extrapolation (R/E) sampling curves (refer to Fig. 2 for the explanations of the abbreviations). Statistical significance was determined with a two-way ANOVA followed with a Tukey’s test. Means with the same letter are not statistically different.

To determine the bacterial taxonomic diversity, richness, and evenness of the bacterial communities within each common bean root genotype grown in two different substrates (soil and vermiculite), alpha diversity indices were calculated (Chao1, Shannon, and Simpson indices). Furthermore, to determine the effects of substrate type, endophytic root compartment, and root genotype, on the diversity indices, two-way ANOVA followed with a Tukey test were performed. The bacterial communities of common bean roots assessed between those from the soil and those from vermiculite showed significant differences for all indices (*P* = 8.47 × 10^−7^, *P* = 1.51 × 10^−6^, *P* = 1.54 × 10^−4^, respectively), except for the evenness (Simpson index) (*P* = 0.379) (Fig. 3A). Moreover, we assessed the effect of genotype on diversity indices of the root-associated bacterial communities from transgenic roots (K599, pK7Neg, and RNAi) and WT roots without significant differences (*P* = 0.0955, *P* = 0.147, *P* = 0.195, *P* = 0.365, respectively). Also, after assessing the diversity (Chao1), richness (Shannon), and evenness (Simpson) indices via rarefaction (interpolation) and extrapolation (R/E) sampling curves analysis, the estimation of Chao1 (order *q* = 0), Shannon (order *q* = 1), and Simpson (order *q* = 2) indices clearly showed significant differences due to the substrate type (Fig. 3B). Next, rarefaction sampling curves estimated for all OTUs and at the genus level showed the same behavior; that is, differences due to the substrate type (Figs. S3A and S3B). Furthermore, a completeness analysis showed that full coverage for all samples was reached below 10,000 reads (Fig. S4A); and coverage analysis for all indices was reached before the extrapolation estimation for all samples (Fig. S4B), which indicates that the differences were not due to a sample size effect.

### Effect of substrate type in common bean roots bacterial communities clustering

**Figure 4:**
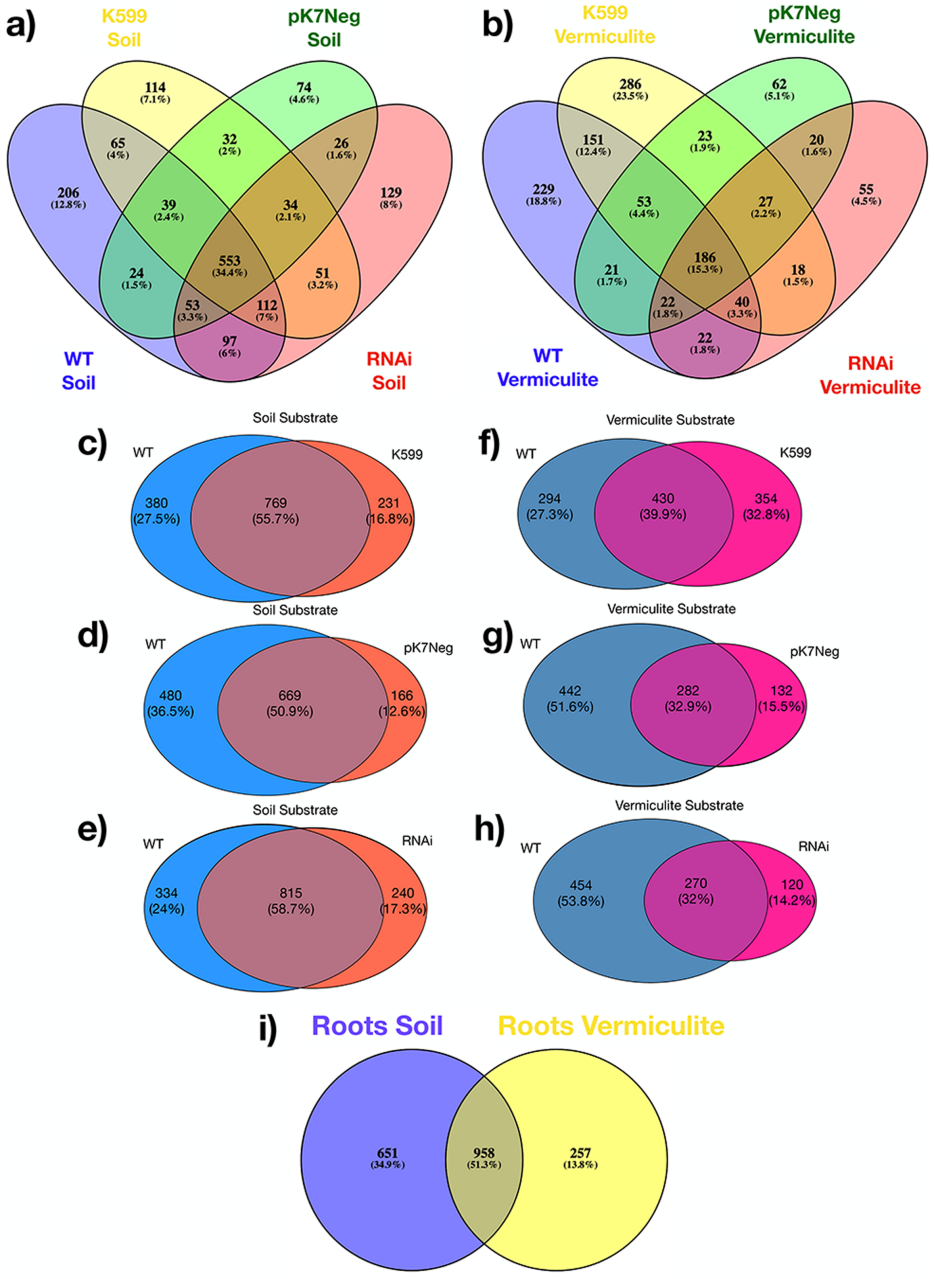
Venn diagrams for OTUs shared among genotypes (transgenics and WT), for both substrates (soil and vermiculite). (A) OTUs shared among all genotypes (WT, K599, pK7Neg, and RNAi) in soil; (B) OTUs shared among all genotypes (WT, K599, pK7Neg, and RNAi) in vermiculite; (C) OTUs shared between K599 transgenic roots and WT roots in soil; (D) OTUs shared between pK7Neg transgenic roots and WT roots in soil; (E) OTUs shared between RNAi transgenic roots and WT roots in soil. (F) OTUs shared between K599 transgenic roots and WT roots in vermiculite; (G) OTUs shared between pK7Neg transgenic roots and WT roots in vermiculite; (H) OTUs shared between RNAi transgenic roots and WT roots in vermiculite. (I) OTUs shared between common bean roots (WT, K599, pK7Neg, and RNAi) in soil and in vermiculite substrates.

A comparative analysis of the common bean roots OTUs shared between all genotypes, shown that a high proportion of OTUs was shared between all root genotypes either for common bean roots grown in soil (67.5%) or common bean roots grown in vermiculite (48.1%). That is, 1,086 OTUs were shared in common bean roots grown in soil, and 583 OTUs were shared in common bean roots grown in vermiculite between all genotypes compared (Fig. 4A). Then, we proceeded to compare the common bean root WT genotype with each common bean root transgenic genotype, grown in soil and vermiculite independently. We found that 50% to 60% of the common bean roots OTUs were shared between common bean roots WT and each common bean root transgenic genotype grown in soil (Fig. 4B), and 32% to 40% of the common bean roots OTUs were shared between common bean roots WT and each common bean root transgenic genotype grown in vermiculite (Fig. 4C). Interestingly, a half (51.3%) of the common bean roots OTUs were shared between soil and vermiculite regardless of the genotype (Fig. 4D).

**Figure 5:**
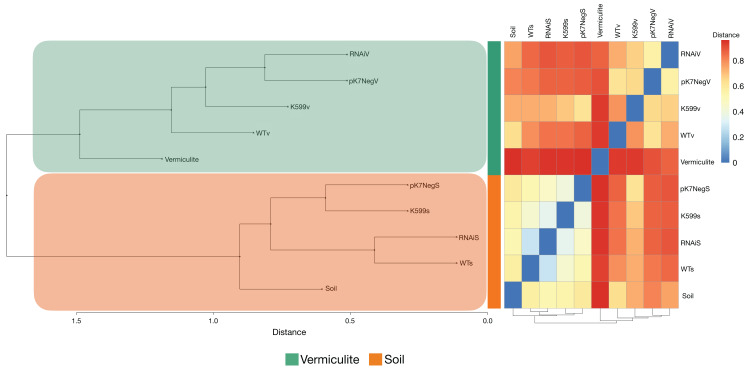
Hierarchical clustering analysis and distance-based heatmap of substrates and the endophytic compartments of roots of common bean. The resulting analysis of Bray–Curtis distances calculated from the relative abundances of the bacterial OTUs associated with the roots of common bean (*P. vulgaris* L.) displayed a clustering based on the type of substrate.

The bacterial communities of the common bean roots for all genotypes were clustered in two distinct groups according to the type of substrate in which the common bean roots were grown (Fig. 5). The bacterial communities in the unplanted substrates were both distinct from those inside the common bean roots grown in these same substrates. Also, the clustering results for the PCoA, PCA, NMDS, CCA, and dbRDA analyses showed that the bacterial communities from the common bean roots were clustered according to the type of substrate and independently of the genotype, and also were similar to those observed in the hierarchical clustering analysis: there were two distinct clusters according to the type of substrate (Figs. S5A–S5E).

The results from the PERMANOVA analysis indicated that the type of substrate was the main factor influencing the bacterial community structures in the common bean roots (*R*^2^ = 0.354, *P* = 0.001) and that the root genotype did not have a significant effect (*R*^2^ = 0.200, *P* = 0.592). Furthermore, the ANOSIM analysis also revealed that the substrate type influences the bacterial community structures (*P* = 0.005) (Fig. S3C). Also, the PERMANOVA analysis for the interaction substrate:genotype (*R*^2^ = 0.228, *P* = 0.042) showed that the substrate was the main factor influencing the common bean roots bacterial community structures regardless of the root genotype (*R*^2^ = 0.201, *P* = 0.066) (Fig. S3D).

Finally, we proceeded to analyze the differential abundance of common bean roots OTUs for all genotypes (WT and transgenics). The analysis for differential abundance between each common bean roots transgenic (K599, pK7Neg, RNAi) genotype and the common bean roots WT genotype per substrate independently, showed that there were slight differences in the number of DAOTUs. The enriched OTUs for K599, pK7Neg, and RNAi roots were 1 (*Blastocatellia*, enriched), 2 (*Enterobacter*, enriched; *Mitsuaria*, depleted), and 1 (*Mitsuaria*, depleted), which represent 0.05%, 0.1%, and 0.05% of the OTUs analyzed, respectively (Figs. S6A–S6C). In RNAi, the more abundant DAOTUs were *Mucilaginibacter*, *Cohnella*, *Paenibacillus glycanilyticus*; and eight that were less abundant: *Aquidulcibacter*, *Rhizobium esperanzae*, *Novosphingobium guangzhouense*, *Burkholderiales*, *Acidovorax*, *Pelomonas puraquae*, *Mitsuaria*, and *Mycoplasmataceae* (representing 0.55% of OTUs analyzed; Fig. S6D). Thus, the limited number of significantly different DAOTUs between all common bean roots genotypes (WT and transgenics) might support why the root genotype is not an influential factor in the structures of the bacterial communities.

## Discussion

The rhizosphere around plant roots contains a high diversity of microorganisms. The plants, through their roots, interact with the microorganism of the rhizosphere, and plant physiology may alter the soil-associated microorganisms and vice versa. The understanding of these interactions seems an essential aspect of the future development of agriculture (Lee et al., 2011; Garcia Ruiz, Knapp & Garcia-Ruiz, 2018). It is thus essential to compare the bacterial diversity in the rhizospheres surrounding the roots of genetically modified and non-genetically modified plants, as well as the bacterial diversity in the endophytic compartments of these last. The first step is to assess the bacterial community assembly to determine whether the bacterial communities of open environments, such as agricultural soils, can induce changes into the endophytic compartments of transgenic plant material (Sahoo & Tuteja, 2013).

The common bean roots colonization studies were restricted only for microorganism symbionts, specifically rhizobia, using gene-specific markers (GFP or DsRed) in these bacterial organisms, and then coupled with the common bean transgenic hairy roots model (Estrada-Navarrete et al., 2006, 2007; Barraza et al., 2015; Estrada-Navarrete et al., 2016). This approach was used to characterize nodule development from the early stages of root colonization up to functional aspects of symbioses, but it has been restricted only to *Rhizobium*-related species (Barraza et al., 2015; Estrada-Navarrete et al., 2016). Moreover, several studies have characterized the microbiome structures of legume nodules with a wide range of approaches, including fully sequencing 16S rDNA and other housekeeping genes, restriction fragment length polymorphism (RFLP) profiling, and next-generation sequencing (454-pyrosequencing platform and Illumina platforms; Yan et al., 2014; Naamala, Jaiswal & Dakora, 2016; Lu et al., 2017; Leite et al., 2017; Sharaf et al., 2019). Those studies applying next-generation sequencing, coupled with bacterial diversity analysis, has been an excellent approach to characterize the bacterial community structures in the legume nodules. We have undertaken the first step in the common bean root bacterial community structure characterization, selecting the common bean transgenic hairy root model to assess the effect of the root genotype and the impact of two different substrates on bacterial community structure.

Based on our results, we found that the bacterial community structures of the common bean roots were affected mostly by the substrate, rather than by the root genotype (WT or transgenic); since the bacterial community structure showed a differential assembly for each substrate. For roots grown in soil substrate, the dominant phylum was *Proteobacteria*, and the second most abundant phyla were *Actinobacteria* and *Acidobacteria*. Whereas for roots grown in vermiculite substrate, the dominant phylum was also *Proteobacteria*, but in higher proportion than in roots grown in soil, and the second most abundant phyla were *Bacteroidetes* and *Firmicutes* (Fig. 2). This bacterial assembly distribution was in accordance with other reports in legume plants for which those phyla were found, with a substrate-dependent differential distribution (Heuer et al., 2002; Sahoo & Tuteja, 2013; Leite et al., 2017; Lu et al., 2017; Sharaf et al., 2019).

Interestingly, we determined an unexpected higher bacterial diversity (observed OTUs and Chao1 index) for common bean roots, either WT or transgenic, compared with substrate either for both soil and vermiculite, suggesting an enrichment phenomenon of bacteria from the substrate inside the common bean roots, but the richness was quite similar (Fig. 3). This phenomenon might be explained by the fact that vermiculite is an inert and nutrient lack substrate (which makes it ideal for experimental assessment in controlled conditions, as we applied in this study). Potting soil has a low pH (acid) and also has a minimal nitrogen content (which provides the environmental and controlled conditions to ensure the bacterial colonization into the common bean roots either WT or transgenic). This enrichment phenomenon was similar to which observed between several common bean roots accessions and bulk native soil (Pérez-Jaramillo et al., 2019). Both substrates soil and vermiculite did not interact with common bean roots, neither WT nor transgenic at any time. However, we were able to determine a substrate-dependent and genotype-independent diversity, richness, and evenness distribution that was even more discrete applying inter- and extrapolation (R/E) sampling curves (for all OTUs and at Genus taxonomic rank level) (Fig. 3B), and applying PERMANOVA and ANOSIM analysis. The high proportion of OTUs shared (~70% in soil, and ~50% in vermiculite) between all common bean root genotypes (WT and transgenics) (Fig. 4). The hierarchical clustering and bi-clustering (heatmap) analyzes of the Bray-Curtis dissimilarity distance matrix (Fig. 5) and the dimensionality reduction or clustering analyzes (PCoA, PCA, NMDS, CCA, and dbRDA) (Fig. S5) showed the same trend: a substrate-dependent distribution of the bacterial community structures of the common bean roots. And finally, the differential abundance of OTUs (DAOTUs) contrasting analyzes between WT and each transgenic genotype per substrate (<1%) also support the genotype-independent distribution of the bacterial community structures (Fig. S6). The WT and transgenic roots genotypes showed a highly similar bacterial communities with small variations. Altogether, the common bean root genotypes did not have a significant influence on the root inner bacterial community structures and strongly suggest that the substrate, which is the main source of nutrients for both the bacterial organisms and plants, is the main factor that shapes the root inner bacterial community. This is a similar conclusion reached for previous reports in *P. vulgaris* and another legume (Leite et al., 2017; Pérez-Jaramillo et al., 2017, 2019).

## Conclusions

The bacterial community structures of common bean roots with four different genotypes were characterized, and all root genotypes showed a direct and significative effect of the substrate type where those roots were grown. This gave rise to suggest that the distribution of the bacterial community structures in this plant organ was modeled following a substrate-dependent pattern. Interestingly, the common bean root genotype did not play as a significant factor that might influence on to the distribution of the bacterial community structures. These give us the guidance to develop efficient, low cost, and ecofriendly biofertilizers with bacterial consortia based on the physicochemical features and nutrient availability of the substrate to enhance the crop production and reduce the environmental impact by decreasing the agrochemical usage in the crop fields.

## Supplemental Information

10.7717/peerj.9423/supp-1 Supplemental Information 1 Generation of transgenic hairy roots in composite common bean plants. (a-b) Fourteen days after infection with *Agrobacterium rhizogenes* the hairy roots were formed, and the primary root was cut off. (c-e) Transgenic hairy roots. (c) K599, (d) pK7Neg vector, (e) PvTRX1h-asRNA (RNAi) Scale bar: 80 µm.

10.7717/peerj.9423/supp-2 Supplemental Information 2 Distribution of the sequence reads at the taxonomic rank of domains.

10.7717/peerj.9423/supp-3 Supplemental Information 3 Rarefaction sampling curves and analysis of similitude and interaction. (a) Rarefaction sampling curves without extrapolation for all OTUs. (b) Rarefaction sampling curves without extrapolation at the genus level. (c) Analysis of similitude (ANOSIM) with the substrate type (soil, vermiculite) as factor, for all samples. (d) PERMANOVA bar plot of the interaction factors (substrate and genotype).

10.7717/peerj.9423/supp-4 Supplemental Information 4 Completeness and coverage estimations. (a) Completeness estimation with inter- and extrapolation methods for all samples. (b) Coverage estimation with inter- and extrapolation methods for all samples (q = 0, Chao1 index; q = 1, Shannon index; q = 2, Simpson index).

10.7717/peerj.9423/supp-5 Supplemental Information 5 Multivariate analysis. (a) Principal coordinates analysis (PCoA); (b) Principal component analysis; (c) Non-metric multidimensional scaling (NMDS) analysis; (d) Constrained correspondence analysis (CCA); (e) Distance-based redundancy analysis (dbRDA).

10.7717/peerj.9423/supp-6 Supplemental Information 6 Comparison of significantly differentially abundant bacterial OTUs between the transgenic lines of common bean (*P. vulgaris* L.; K599, pK7Neg, and RNAi) and the wild type. (a) Comparison of K599 transgenic roots, (b) pK7Neg transgenic roots, and (c) RNAi transgenic roots to WT control roots in Soil; (d) Comparison of RNAi transgenic roots to WT control roots in Vermiculite. Benjamini-Hochberg’s false discovery rate was used to adjust the significance levels for multiple testing (*P* < 0.05). Each bar represents an OTU labeled at the lowest taxonomic rank, and colored based on the associated phylum.

## Additional Information and Declarations

### Data Availability

The following information was supplied regarding data availability: Sequences are available at NCBI BioProject: PRJNA552113.
