# Supplementary figures and images for: Highly diverse root endophyte bacterial community is driven by growth substrate and is plant genotype-independent in common bean (Phaseolus vulgaris L.)
Source: PeerJ. 2020 Jun 26;8:e9423. doi: 10.7717/peerj.9423 (PMC7323714; doi:10.7717/peerj.9423)

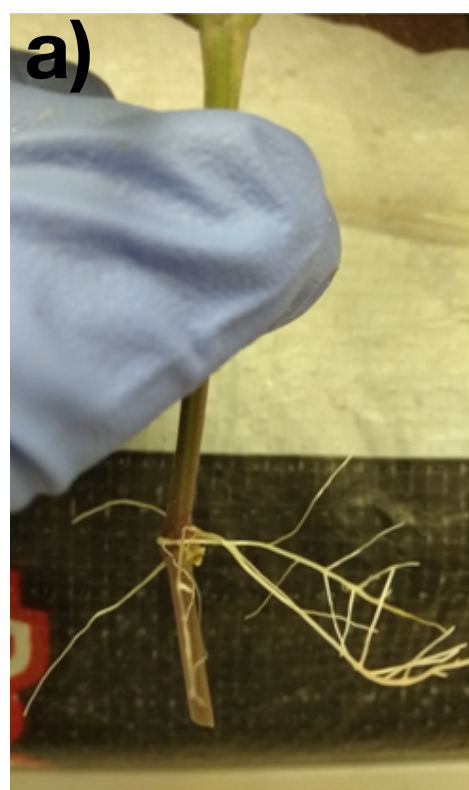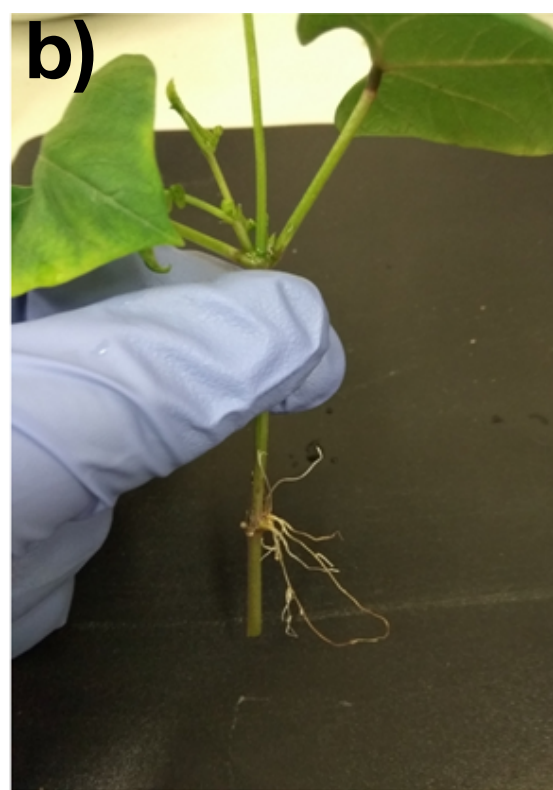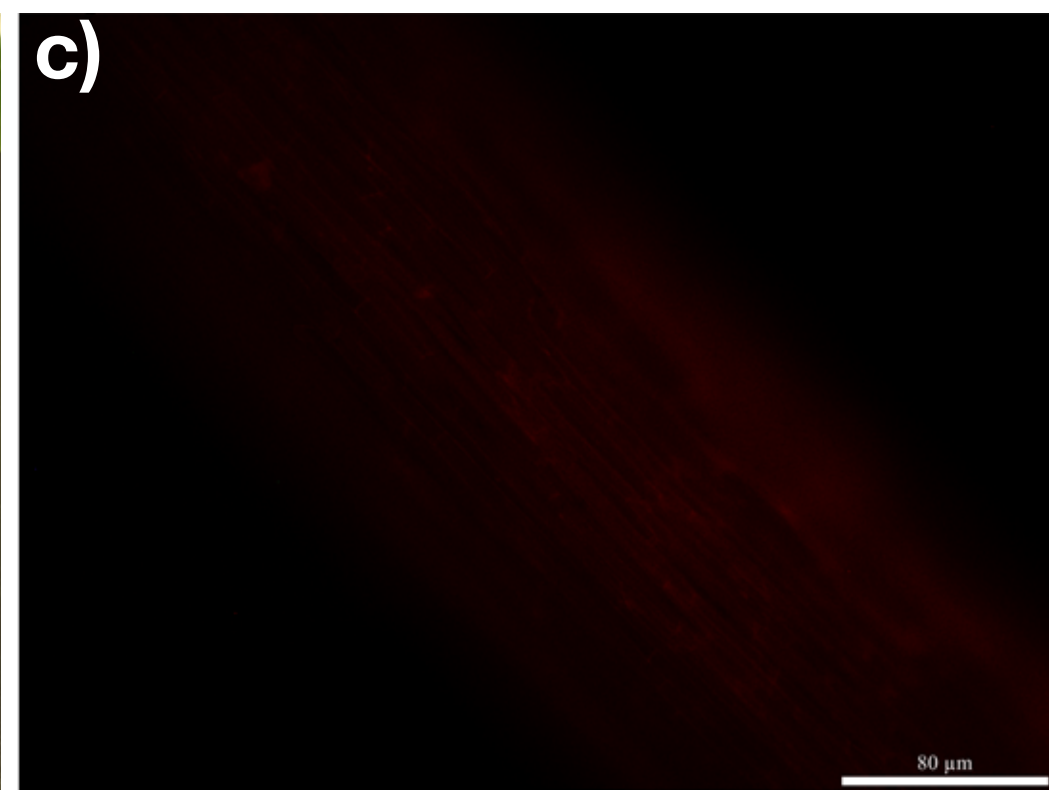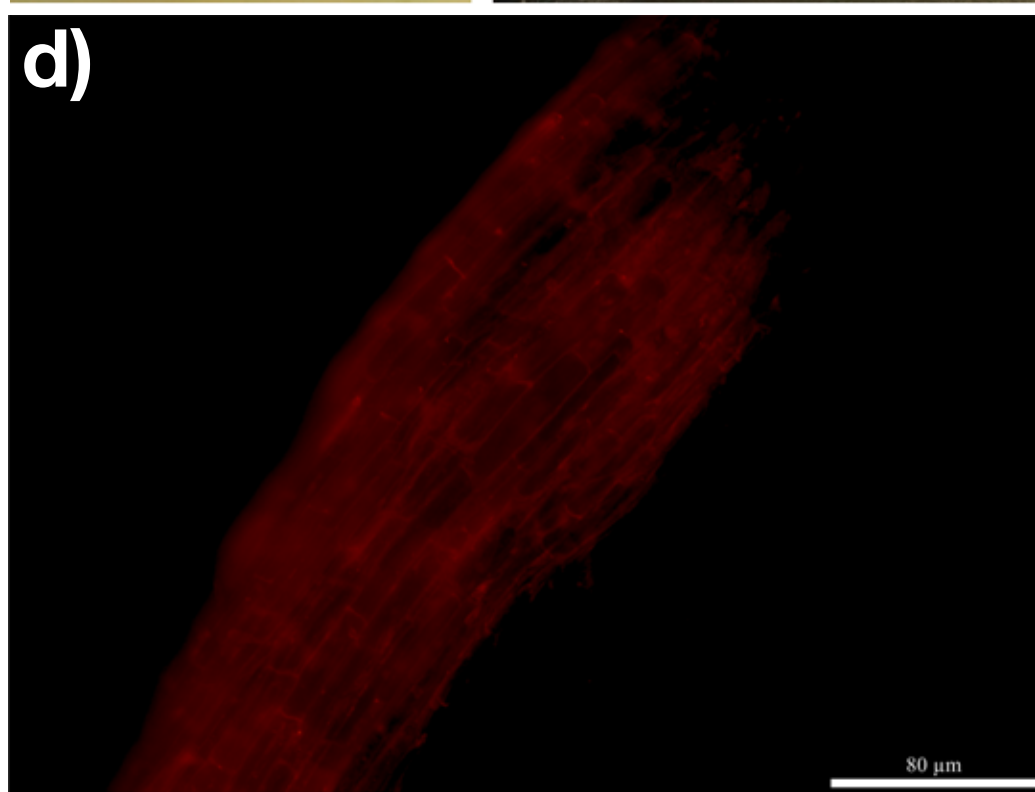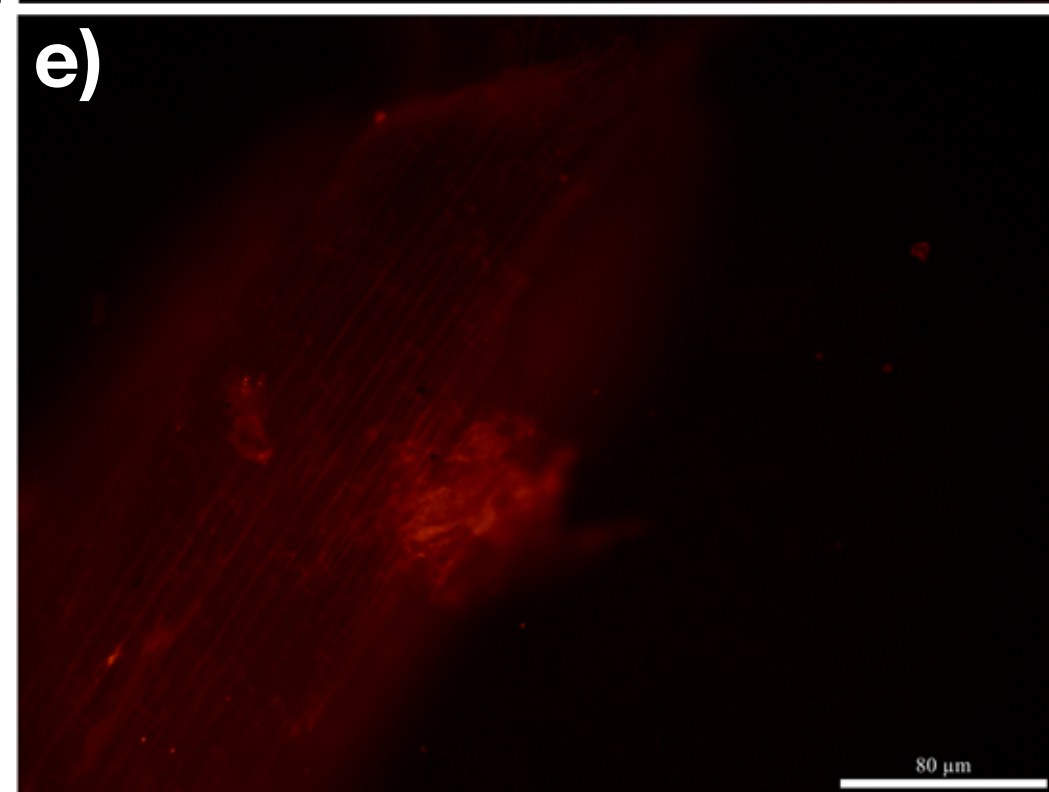

Supplement: Supplemental Information 1 — (a-b) Fourteen days after infection with Agrobacterium rhizogenes the hairy roots were formed, and the primary root was cut off. (c-e) Transgenic hairy roots. (c) K599, (d) pK7Neg vector, (e) PvTRX1h-asRNA (RNAi) Scale bar: 80 µm. [file peerj-08-9423-s001.pdf]

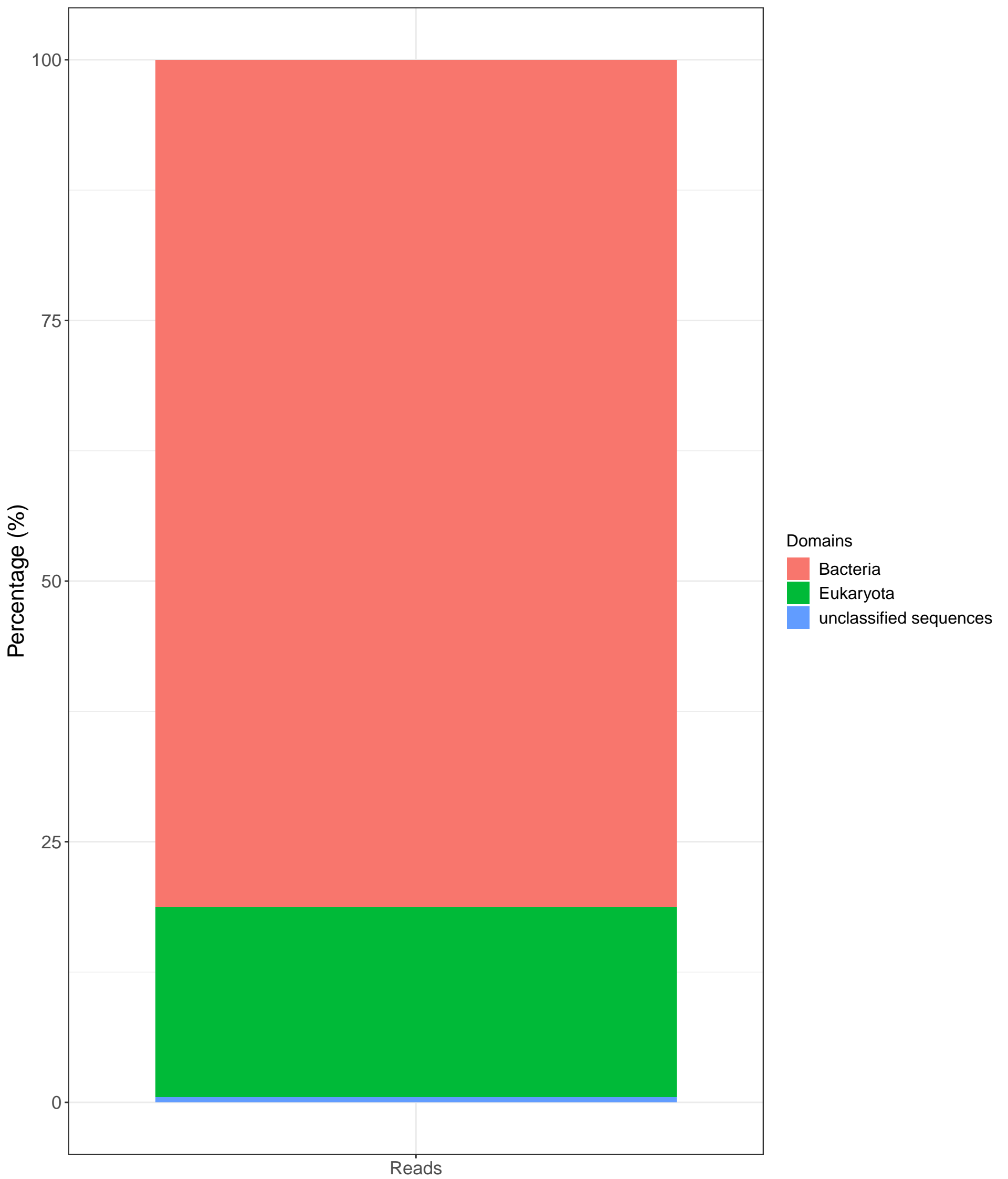

Supplement: Supplemental Information 2 [file peerj-08-9423-s002.pdf]

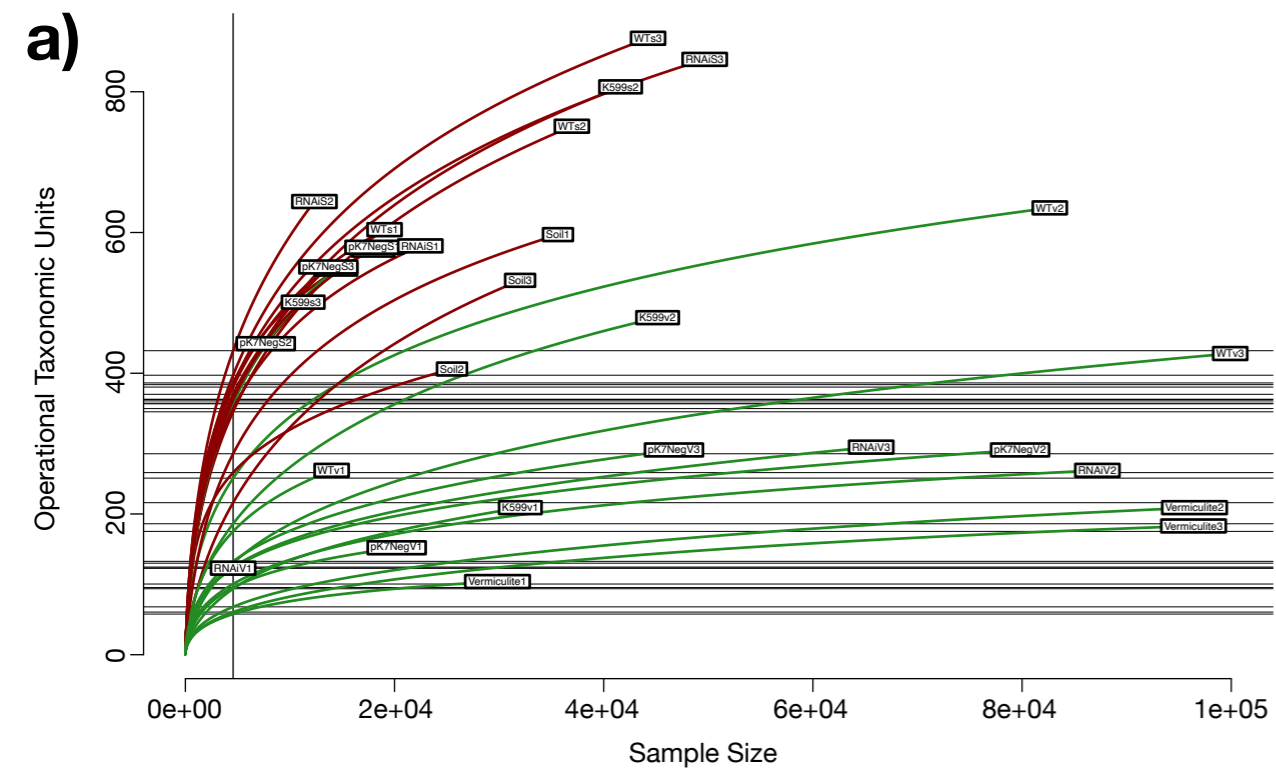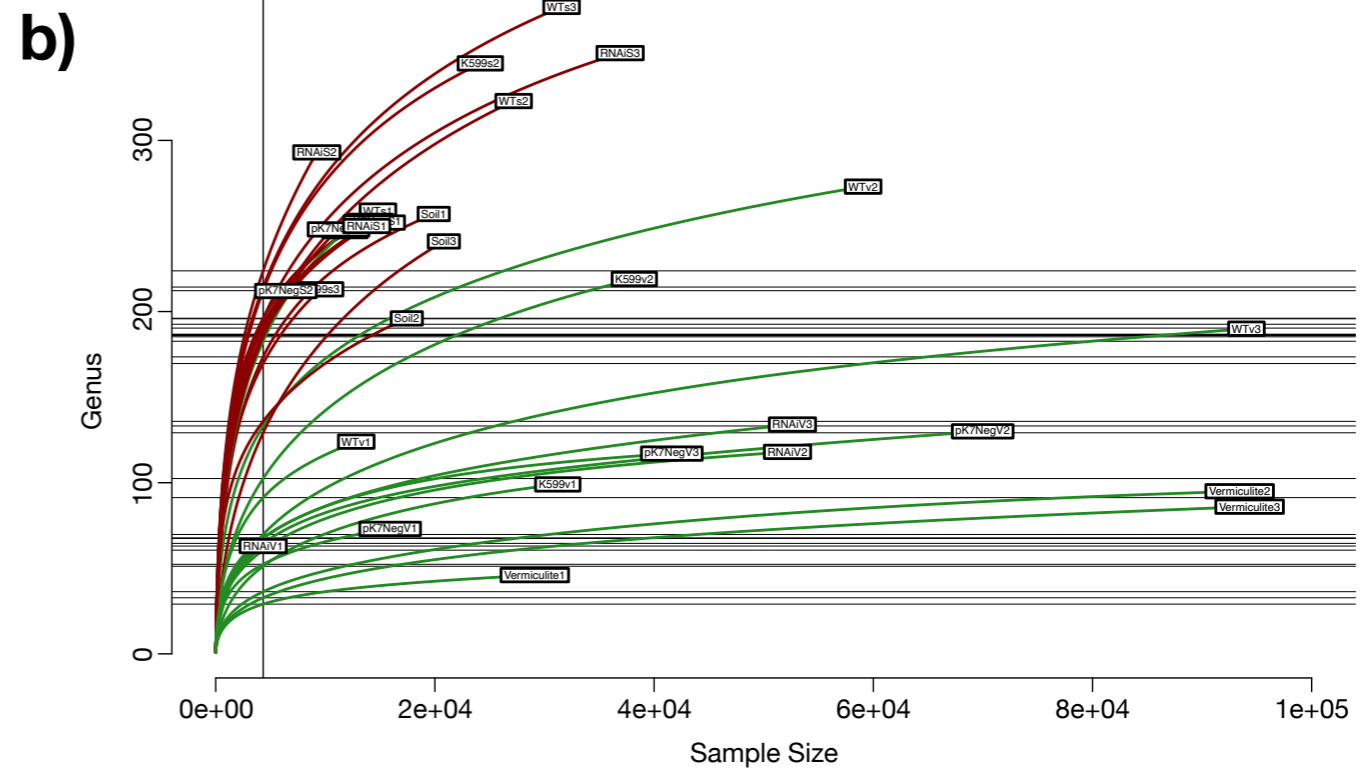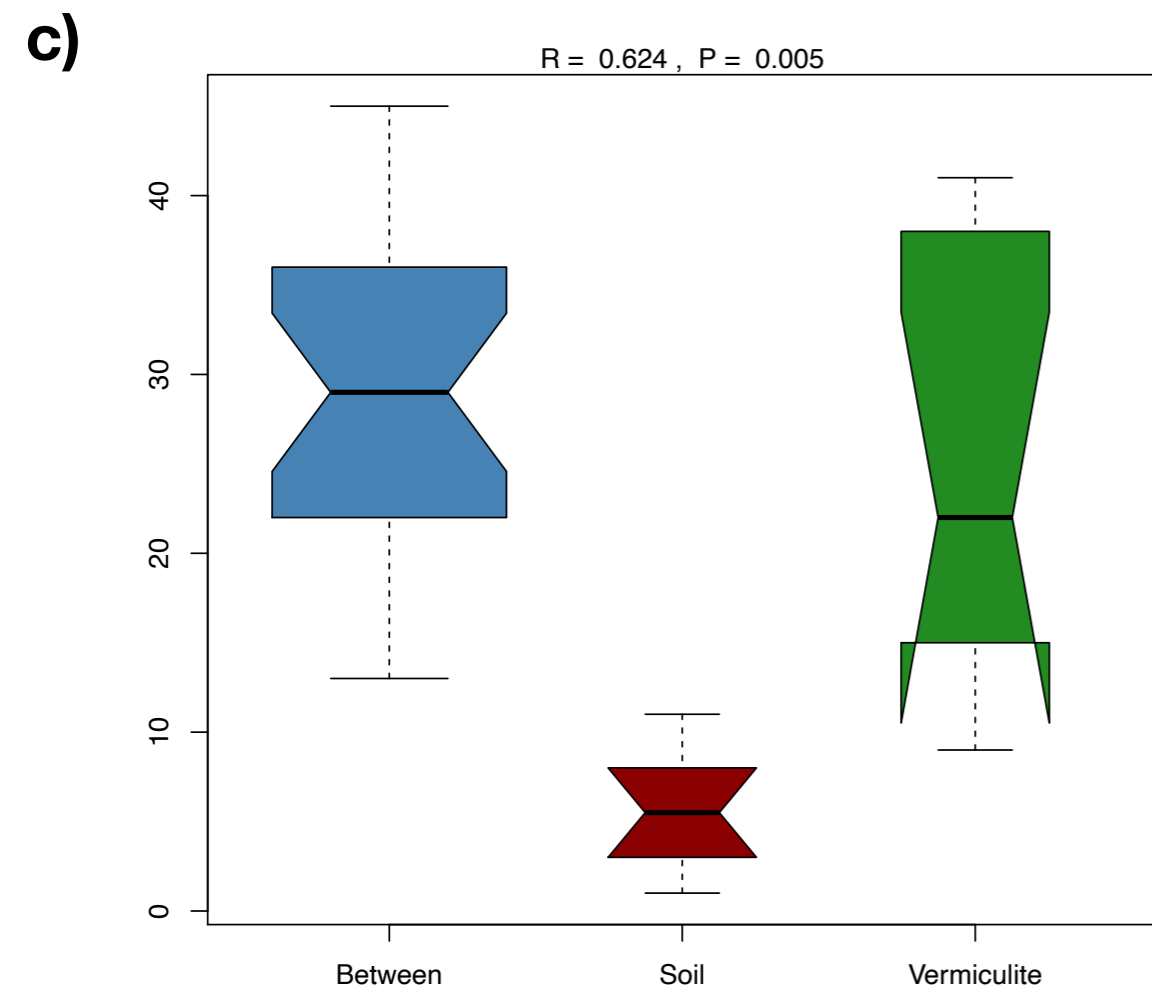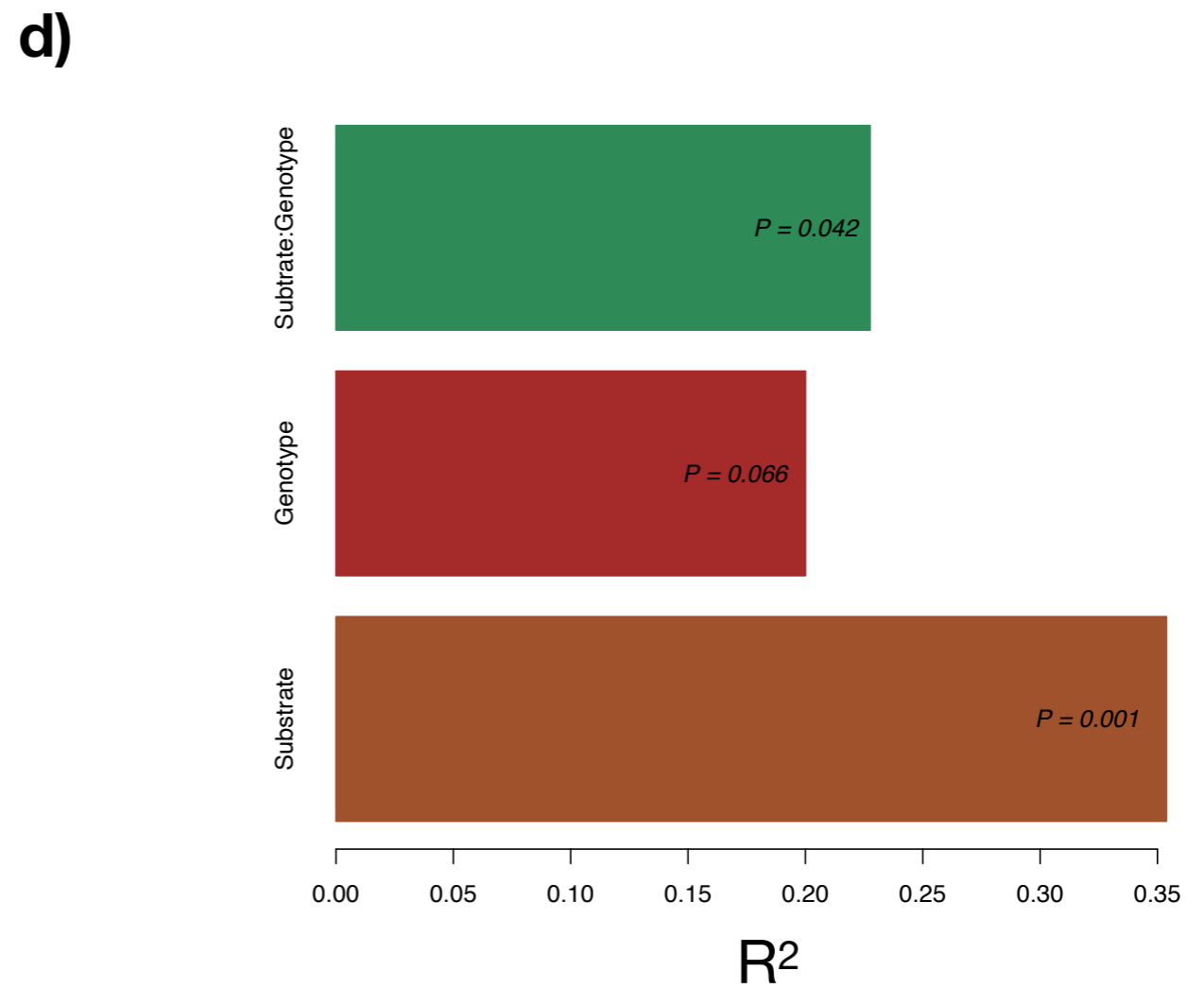

Supplement: Supplemental Information 3 — (a) Rarefaction sampling curves without extrapolation for all OTUs. (b) Rarefaction sampling curves without extrapolation at the genus level. (c) Analysis of similitude (ANOSIM) with the substrate type (soil, vermiculite) as factor, for all samples. (d) PERMANOVA bar plot of the interaction factors (substrate and genotype). [file peerj-08-9423-s003.pdf]

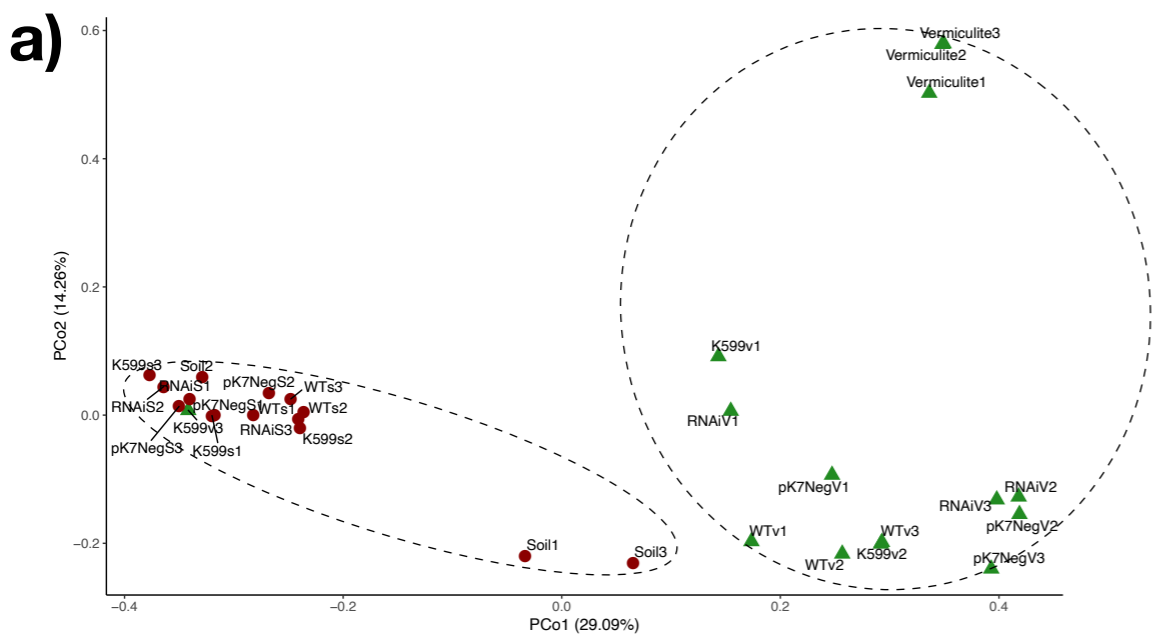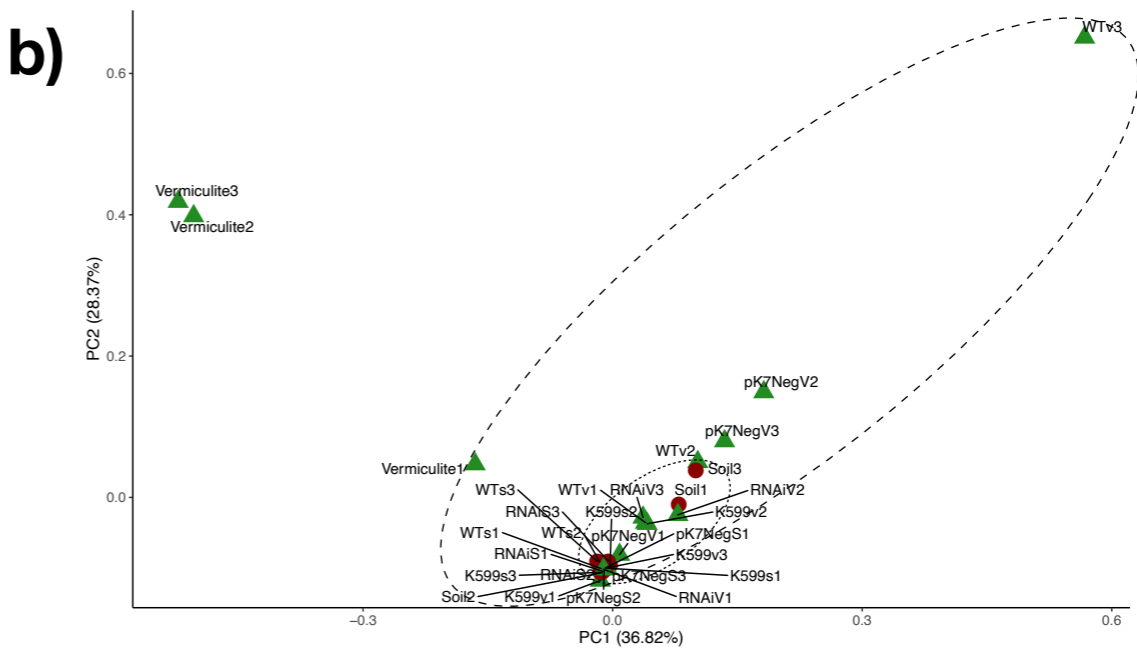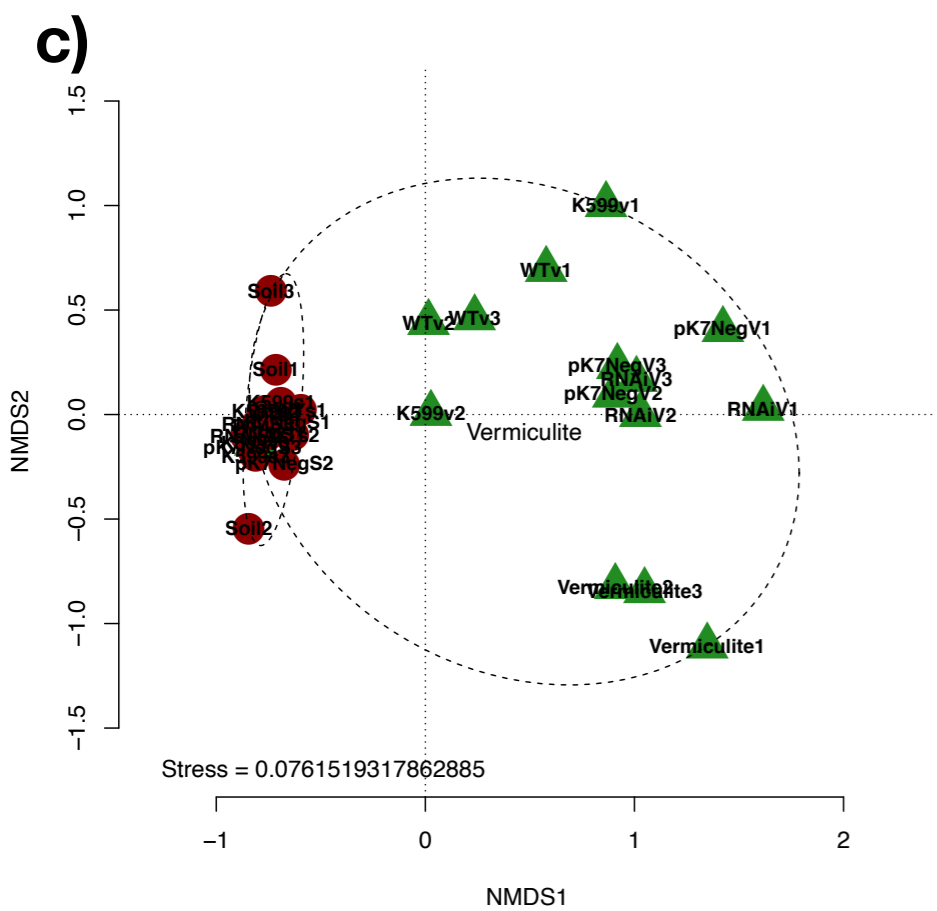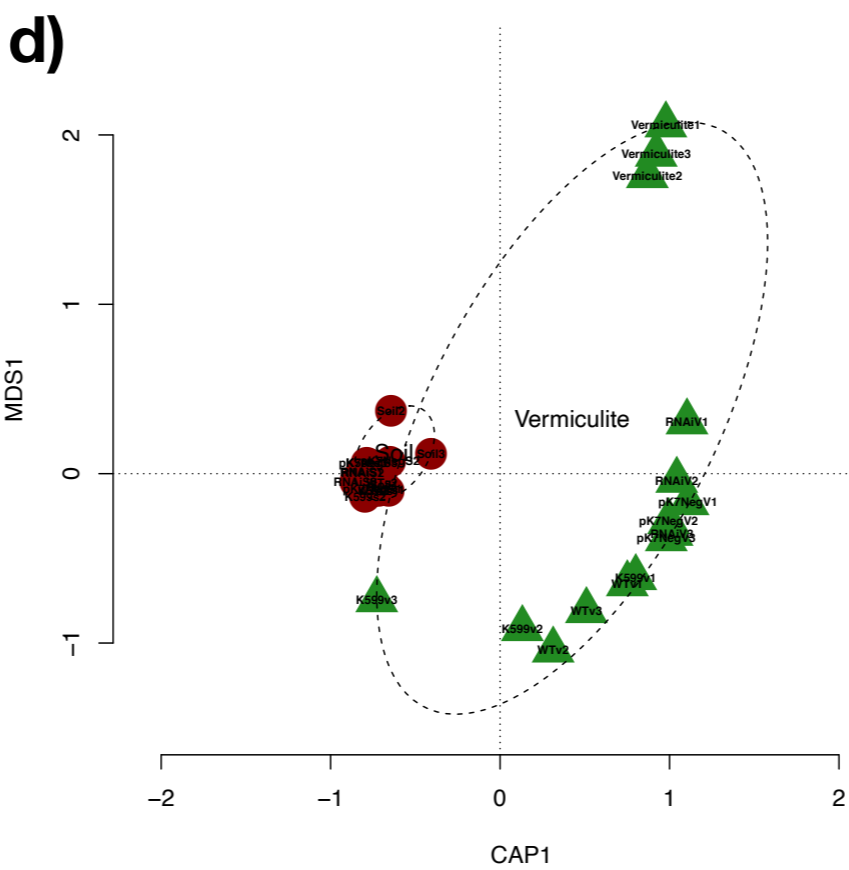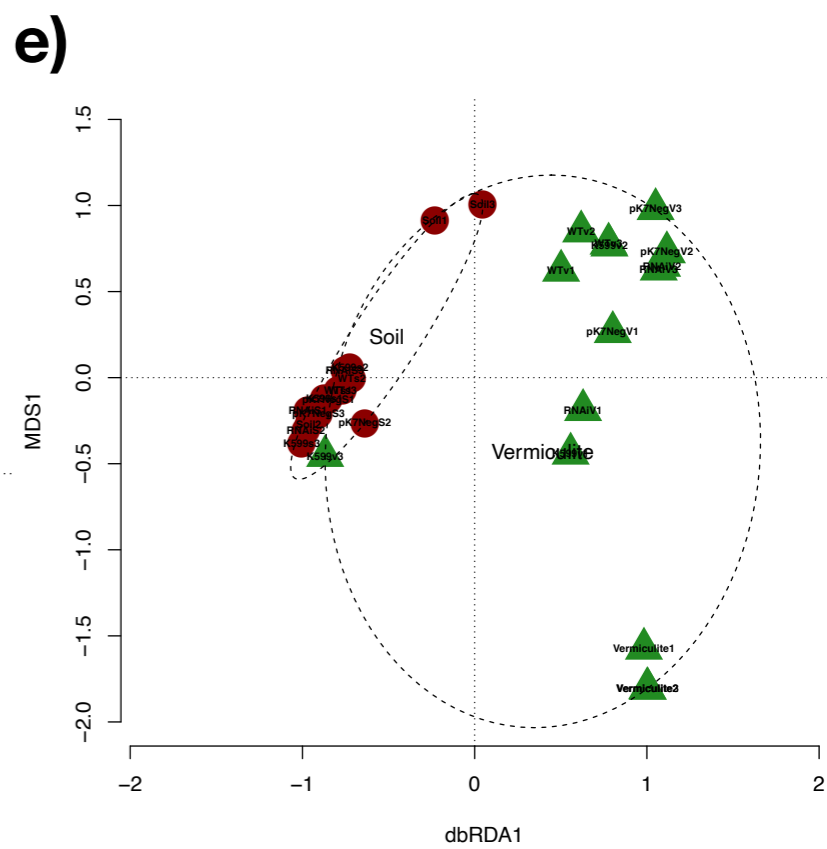

Supplement: Supplemental Information 5 — (a) Principal coordinates analysis (PCoA); (b) Principal component analysis; (c) Non-metric multidimensional scaling (NMDS) analysis; (d) Constrained correspondence analysis (CCA); (e) Distance-based redundancy analysis (dbRDA). [file peerj-08-9423-s005.pdf]
