# Supplementary material for: Highly diverse root endophyte bacterial community is driven by growth substrate and is plant genotype-independent in common bean (Phaseolus vulgaris L.)
Source: PeerJ. 2020 Jun 26;8:e9423. doi: 10.7717/peerj.9423 (PMC7323714; doi:10.7717/peerj.9423)
Supplement: Supplemental Information 4 — (a) Completeness estimation with inter- and extrapolation methods for all samples. (b) Coverage estimation with inter- and extrapolation methods for all samples (q = 0, Chao1 index; q = 1, Shannon index; q = 2, Simpson index). [file peerj-08-9423-s004.pdf]

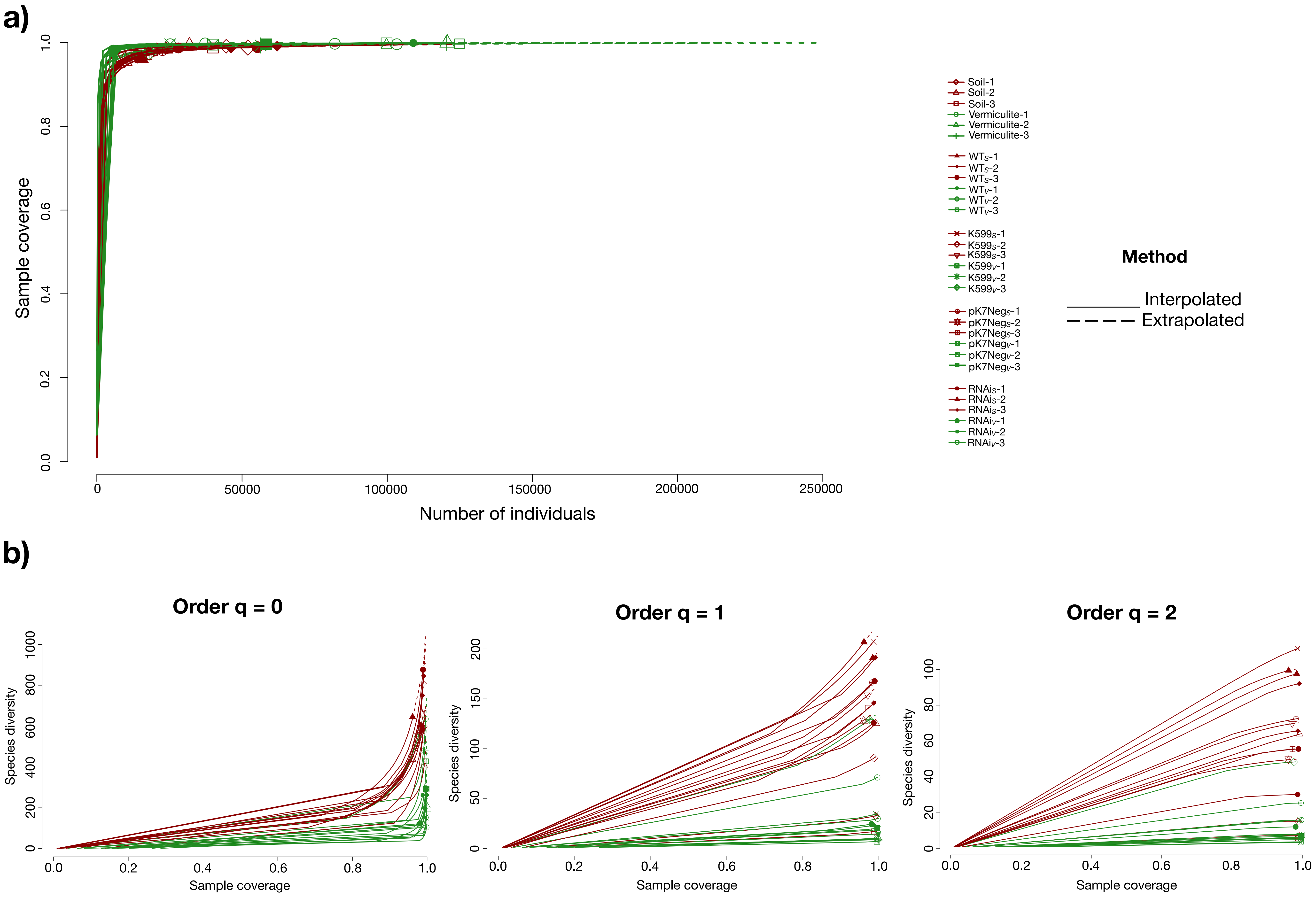

a)
Sample coverage
0.0
0.2
0.4
0.6
0.8
1.0
0
50000
100000
150000
200000
250000
Number of individuals
Soil-1
Soil-2
Soil-3
Vermiculite-1
Vermiculite-2
Vermiculite-3
WTs-1
WTs-2
WTs-3
WTv-1
WTv-2
WTv-3
K599s-1
K599s-2
K599s-3
K599v-1
K599v-2
K599v-3
pK7Negs-1
pK7Negs-2
pK7Negs-3
pK7Negv-1
pK7Negv-2
pK7Negv-3
RNAis-1
RNAis-2
RNAis-3
RNAiv-1
RNAiv-2
RNAiv-3
Method
Interpolated
Extrapolated
b)
Order q = 0
Species diversity
0
200
400
600
800
1000
0.0
0.2
0.4
0.6
0.8
1.0
Sample coverage
Order q = 1
Species diversity
0
50
100
150
200
0.0
0.2
0.4
0.6
0.8
1.0
Sample coverage
Order q = 2
Species diversity
0
20
40
60
80
100
0.0
0.2
0.4
0.6
0.8
1.0
Sample coverage
