# Supplementary material for: Highly diverse root endophyte bacterial community is driven by growth substrate and is plant genotype-independent in common bean (Phaseolus vulgaris L.)
Source: PeerJ. 2020 Jun 26;8:e9423. doi: 10.7717/peerj.9423 (PMC7323714; doi:10.7717/peerj.9423)
Supplement: Supplemental Information 6 — (a) Comparison of K599 transgenic roots, (b) pK7Neg transgenic roots, and (c) RNAi transgenic roots to WT control roots in Soil; (d) Comparison of RNAi transgenic roots to WT control roots in Vermiculite. Benjamini-Hochberg’s false discovery rate was used to adjust the significance levels for multiple testing (P < 0.05). Each bar represents an OTU labeled at the lowest taxonomic rank, and colored based on the associated phylum. [file peerj-08-9423-s006.pdf]

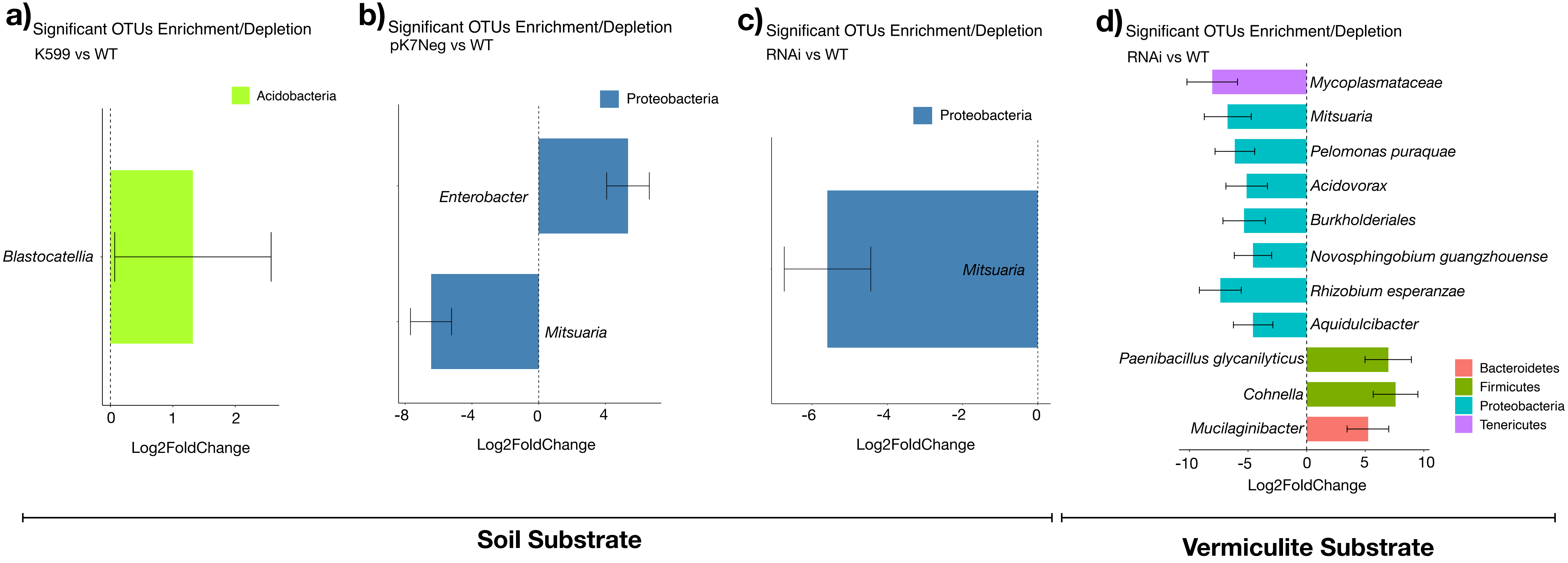
a)
Significant OTUs Enrichment/Depletion
K599 vs WT
Acidobacteria
Blastocatellia
0
1
2
Log2FoldChange
b)
Significant OTUs Enrichment/Depletion
pK7Neg vs WT
Proteobacteria
Enterobacter
Mitsuaria
-8
-4
0
4
Log2FoldChange
c)
Significant OTUs Enrichment/Depletion
RNAi vs WT
Proteobacteria
Mitsuaria
-6
-4
-2
0
Log2FoldChange
d)
Significant OTUs Enrichment/Depletion
RNAi vs WT
Mycoplasmataceae
Mitsuaria
Pelomonas puraquae
Acidovorax
Burkholderiales
Novosphingobium guangzhouense
Rhizobium esperanzae
Aquidulcibacter
Paenibacillus glycanilyticus
Cohnella
Mucilaginibacter
Bacteroidetes
Firmicutes
Proteobacteria
Tenericutes
-10
-5
0
5
10
Log2FoldChange
Soil Substrate
Vermiculite Substrate
